# On the nature of spin reorientation transition thermal hysteresis in NiO(111)/Fe(110) bilayers

**DOI:** 10.1038/s41598-025-07541-1

**Published:** 2025-07-01

**Authors:** E. Świerkosz, A. Kwiatkowski, M. Szpytma, W. Janus, M. Zając, P. Dróżdż, E. Oleś, A. Kozioł-Rachwał, T. Ślęzak, M. Ślęzak

**Affiliations:** 1 AGH University of Krakow, Kraków, Poland; 2 Elettra-Sincrotrone Trieste S.C.p.A, Basovizza, Trieste, Italy; 3 Present Address: Institut de Ciència de Materials de Barcelona (ICMAB-CSIC), Campus de la UAB, Bellaterra, 08193 Spain; 4 National Synchrotron Radiation Centre SOLARIS, Jagiellonian University, Kraków, Poland

**Keywords:** Magnetic anisotropy, Hysteresis, Ferromagnet, Antiferromagnet, Spintronics, Condensed-matter physics, Spintronics, Surfaces, interfaces and thin films

## Abstract

We report on temperature-driven in-plane 90° magnetization switching in NiO(111)/Fe(110) bilayer epitaxially grown on a W(110) single crystal, investigated using magneto-optical Kerr effect and X-ray magnetic circular and linear dichroism measurements. As the temperature varies, an abrupt switching of the easy axis between the in-plane Fe[001] and Fe$\left[ {1\overline{{{\text{1}}}} 0} \right]$ crystallographic directions is observed. In the temperature range of approximately 210–285 K, a thermal hysteresis region appears, where two energy minima coexist at a given temperature. Our experimental findings are supported by phenomenological modeling. Simulations incorporating temperature-dependent anisotropy constants successfully reproduce the key features of the observed phenomenon, most notably the temperature-driven hysteresis of ferromagnetic magnetization switching. The spin reorientation transition in both exchange-coupled ferromagnetic and antiferromagnetic layers is driven by the interplay between magnetocrystalline and interfacial magnetic anisotropies in the ferromagnet, which stabilizes specific magnetization orientation at given temperature.

**Supplementary Information:**

The online version contains supplementary material available at 10.1038/s41598-025-07541-1.

## Introduction

Hysteresis, a phenomenon in which the state of a system depends not only on current conditions but also on its history, was first introduced in 1882 by J. A. Ewing to describe the behavior of magnetic materials^1^. Since then, it has been observed in various natural, physical, non-physical, and engineering systems, where the response to changes in inputs is delayed or depends on the system’s past states including ocean and climate science^2^, economics^3^, biology^4^ or mechanics^5^. Among the variety of hysteresis examples, temperature-induced hysteresis phenomena appears particularly interesting, both due to their applications in everyday life (e.g., thermostats controlling heating and cooling systems) and their potential negative impacts such as in the case of the giant magnetocaloric effect in magnetic-cooling machines^6^. Thermal hysteresis arises naturally in various magnetic systems, ranging from geophysics^7^ to skyrmionic materials^8^. Magnetic thin film architectures can serve as good model systems exhibiting thermal hysteresis, as theoretically predicted for spin reorientation transition (SRT) phenomena^9,10^. Nevertheless, corresponding experimental reports are very rare and limited to thermal hysteresis of SRT of ferromagnetic components in either purely ferromagnetic^11,12^ or ferromagnet/antiferromagnet (FM/AFM)^13–15^ systems. A separate class of systems where thermal hysteresis has also been reported includes more complex materials and alloys that exhibit temperature-induced structural or FM-AFM phase transitions, such as MnBi^16^, FeRh^17–19^ or MnRh^20^.

The aim of this report is to provide experimental evidence and a phenomenological description of thermal hysteresis in a simple, model-like SRT occurring in FM/AFM epitaxial bilayers. The subject of our study is the epitaxial NiO(111)/Fe(110) bilayer grown on a W(110) single-crystal surface. This choice ensures that, as the temperature changes, the system does not undergo any structural or magnetic phase transitions other than the temperature-induced spin reorientation, which is the primary focus of this study. The structure of both antiferromagnetic NiO and ferromagnetic Fe sublayers is practically temperature independent at 80–350 K studied range. Importantly, the system is also still relatively far from its both ordering temperatures, as the Curie temperature of Fe is bulk-like (~ 1040 K) while the Néel temperature of NiO(111) layer is ∼400 K^21^. For this reason, theoretically predicted, temperature induced magnetic rearrangements at the FM/AFM interface in the vicinity of ordering temperature(s)^22^ are neither expected, nor observed.

As we show in the following sections, the sole driving force behind the temperature-induced switching of magnetic moments in both exchange-coupled FM and AFM components is the well-established in-plane to in-plane 90º SRT in ferromagnetic Fe(110). This phenomenon has been previously reported by several groups in bare Fe(110) films^23,24^ as well as in Fe(110) capped with non-magnetic^25,26^, ferromagnetic^27–29^, and more recently, antiferromagnetic CoO^30,31^ and NiO^21,32,33^ layers. However, when the Fe thickness is close to the critical SRT value, the magnetic anisotropy (MA) becomes low enough to allow temperature-driven rotation of magnetic moments from the Fe[1̅10] to the Fe[001] in-plane direction. In purely ferromagnetic systems, this results from the specific temperature dependence of Fe MA constants^34^. It is interesting to follow temperature induced SRT and its thermal hysteresis in AFM/FM bilayers. In these systems one can expect effects that can be classified in two general groups. In one of them, for exchange biased AFM/FM bilayers, depending on magnetic anisotropy and applied field cooling procedure, AFM can play a master role and govern the magnetization of adjacent FM. For example, exchange bias along chosen axis acts as an additional (unidirectional) magnetic anisotropy and below some critical temperature it can make FM magnetization to rotate independently on its own, intrinsic MA. The second group, on which we focus in the present report, includes exchange bias-free systems where much simpler scenario is expected. Specifically, rotatable spins of AFM should in such a case follow the reorientation of magnetization in FM, which now plays a dominant role in the system. Such a straightforward temperature-induced SRT driven solely by the MA of the FM layer can indeed only be realized in an exchange bias-free FM/AFM system with a magnetically soft antiferromagnet. This is why we chose the NiO(111)/Fe(110) system for this study instead of the alternative CoO(111)/Fe(110) system, where the strong temperature dependence of the exchange bias dominates over the weakly temperature-dependent MA of Fe^35^.

## Results and discussion

In order to allow temperature-driven rotation of magnetic moments from the Fe[11̅0] to the Fe[001] in-plane direction one has to ensure that the Fe thickness is close to the critical SRT value and thus the MA of Fe becomes low enough. The critical SRT thickness, which for uncovered Fe(110) films varies from 60 Å up to 130 Å depending on the preparation recipe, can be drastically lowered (even down to ∼10–80 Å range) in the case of Au/Fe^36^ bilayers, moderately modified by adsorption of gases on the surface or extremely enlarged (> 500 Å) in case of metastable bcc Co and Fe/Co overlayers^27^ on Fe(110). In the present report, for NiO(111)/Fe(110) bilayers the critical SRT thickness at 300 K is ~ 90 Å. In the Supplementary Material (Fig. 5) we also present MOKE results for AFM-free Au(111)/Fe(110) bilayers on W(110), where the most suitable Fe thickness for temperature induced SRT study was tuned to 80 Å.

**Fig. 1:**
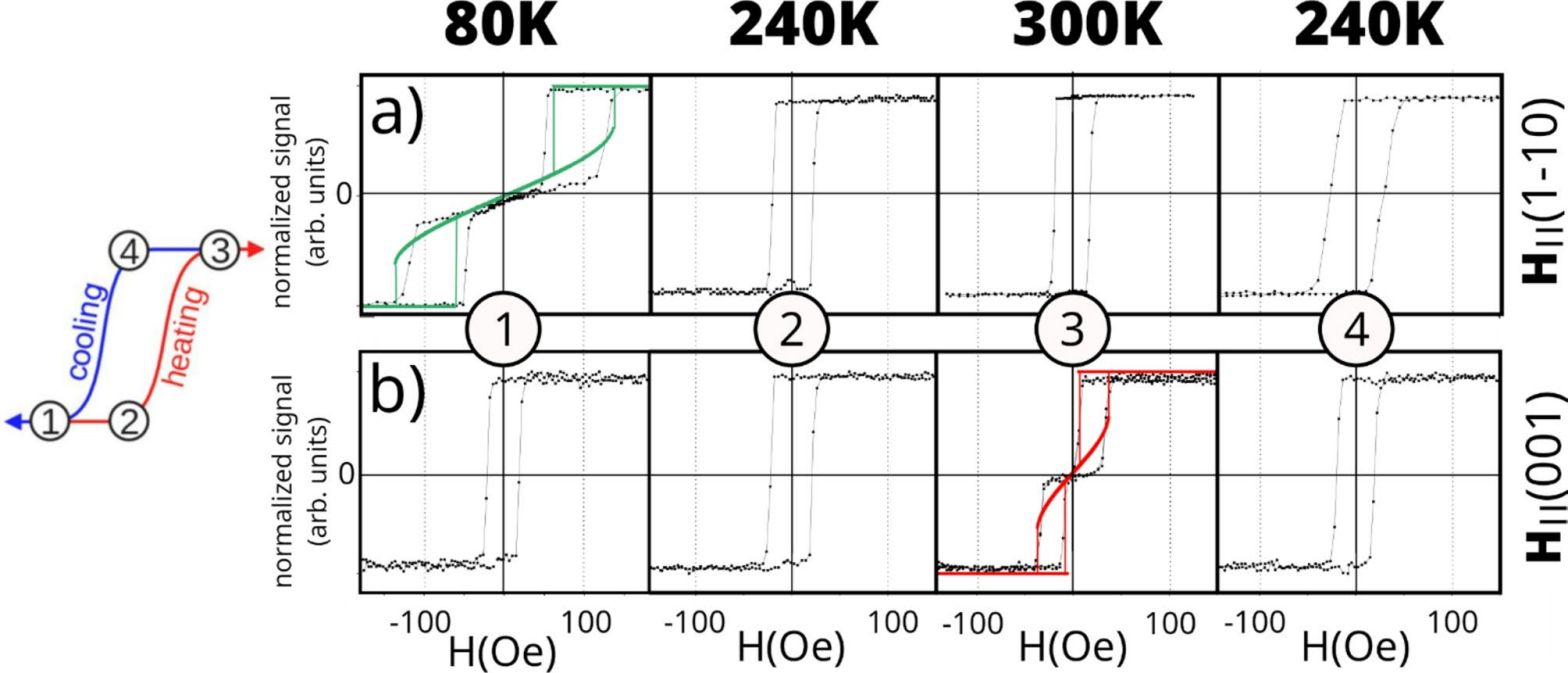
Magnetic hysteresis loops for a 90 Å Fe film covered by 40 Å thick NiO overlayer, as measured at 300 K, 240 K, and 80 K for two complementary LMOKE geometries. The top row (a) corresponds to the external magnetic field applied along in-plane Fe[1–10] crystallographic direction, the data set on the right (b) includes data acquired for the magnetic field applied along Fe[001] direction. Additionally, two exemplary simulated magnetization hysteresis loops are shown, based on which temperature dependent anisotropy constants have been determined.

In Fig. 1 we show exemplary magnetic hysteresis loops for 90 Å Fe film covered by 40 Å thick NiO overlayer, as measured at 300 K, 240 K and 80 K temperatures, for two complementary Longitudinal Magnetooptic Kerr Effect (LMOKE) geometries, namely with the external magnetic field applied along Fe[1–10] (Fig. 1(a)) and Fe[001] (Fig. 1(b)).

**Fig. 2:**
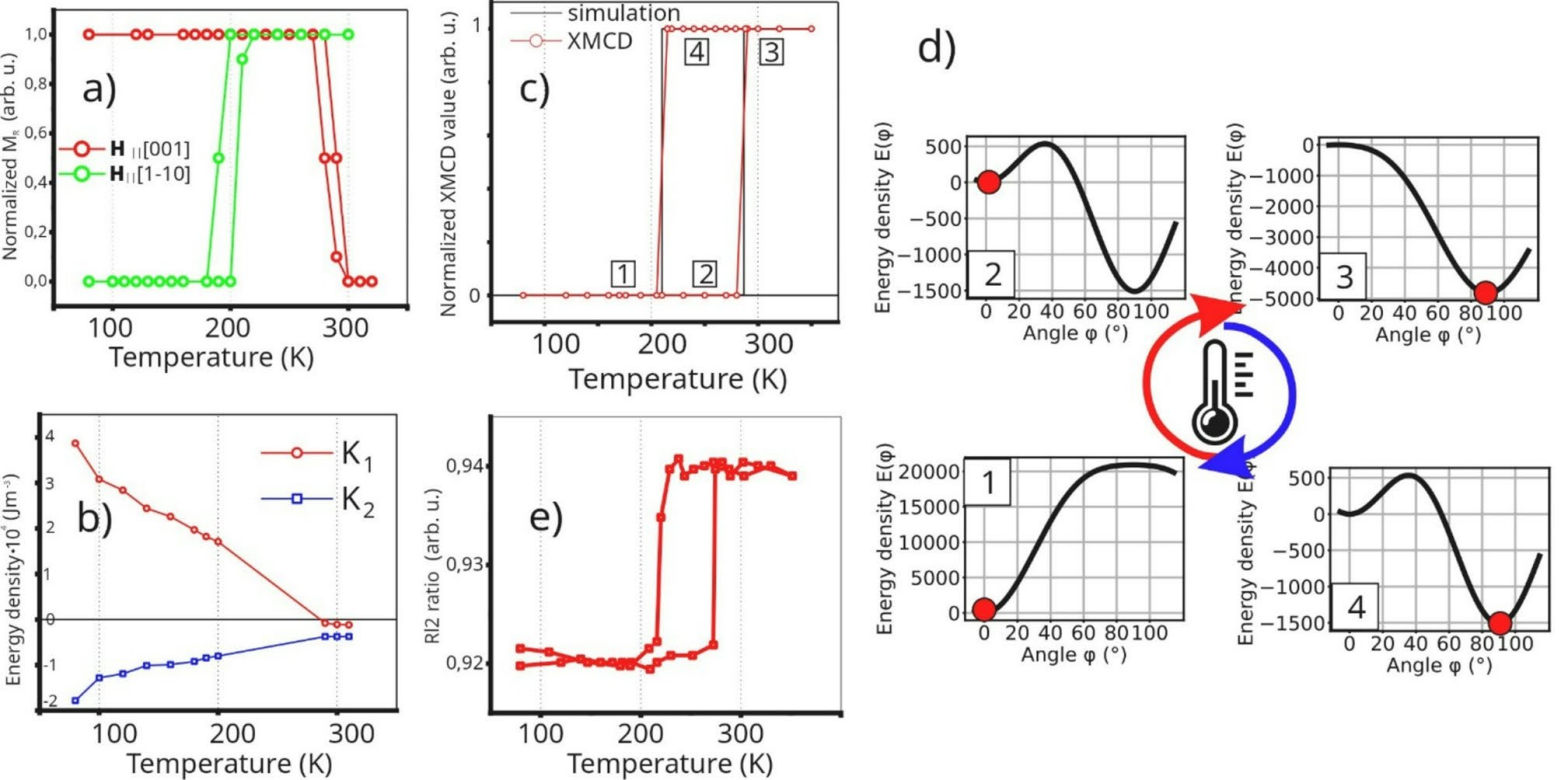
(a) Normalized magnetization in remanence state M_R_ as determined from magnetic hysteresis loops, as a function of temperature, for two LMOKE geometries. (b) Magnetic anisotropy constants K_1_ and K_2_ as derived from comparison of simulations with LMOKE loops (open symbols). Continuous function approximations of K_1_(T) and K_2_(T) dependencies are shown with solid lines. (c) Results of simulations along with XMCD data. 2(d) The corresponding E($\:{\uptheta\:},\:\text{T}$) dependencies at representative temperatures. Each panel depicts a schematic energy curve with a red dot indicating the equilibrium magnetization direction. The magnetization progresses from states 1–2 at lower temperatures to states 3–4 at higher temperatures, reflecting how the interplay between magnetocrystalline and interfacial anisotropies of Fe reshapes the potential minima as temperature changes. Consequently, the system undergoes a 90° reorientation transition in its easy axis, consistent with the observed experimental data. (e) XMLD as quantified by the R_L2_ ratio temperature dependence (see ‘Methods’ for definition R_L2_ ratio).

From the results obtained in both geometries, it is clear that a temperature induced SRT takes place between 80 K and 300 K. At low temperature the easy axis of Fe is oriented along [001] direction, as indicated by typical square hysteresis loop for **H** || [001] and hard-like magnetic hysteresis loop, with zero magnetization in remanent state for **H** || [1–10]. At 300 K, the character of the magnetic hysteresis loops is reversed between two LMOKE geometries, which means that at high temperature Fe prefers to be spontaneously magnetized along in-plane [1–10] direction, in agreement with results concerning temperature induced SRT in uncovered Fe(110)^34^ and W(110)/Fe(110)/W(110) films^37^. The character of hard-axis ‘stepped-like’ loops with characteristic jumps of magnetization should be explained. As the external magnetic field increases, initially the magnetization of Fe is continuously rotating towards its hard axis and at some critical point (saturation field) it suddenly jumps to align with **H**. This effect often (but not always) displays hysteresis, i.e. as magnetic fields decreases the switching field can be smaller as compared to ascending branch. Value of switching field together with the slope of hysteresis loop during the initial continuous rotation of Fe magnetization precisely define the in-plane magnetic anisotropy in the system, as shown in pioneering work of Elmers and Gradmann^38^. Such characteristic ‘stepped-like’ magnetic hysteresis loops are not restricted to Fe/W(110)^28^ but were also observed in other systems with strong uniaxial anisotropy, for example induced by atomic steps for Fe on vicinal Ag(1,1,10) surface^39^. The critical temperatures at which spin reorientation transitions occur are 210 K during cooling and 280 K during heating, as evidenced by the temperature dependence of the remanent magnetization shown in Fig. 2(a). The transient points observed in Fig. 1(a) and 1(b) at 240 K and at 210–280 K range in Fig. 2(a) may at first glance appear to be inconsistent with each other, however the occurrence of square, easy-like magnetic hysteresis loops for both LMOKE geometries and during both cooling and heating procedures is characteristic for hysteresis region of SRT and is an inherent property of such field-assisted tracing of temperature SRT. This aspect is discussed in more detail later in the text. In Fig. 1(a), (b) two exemplary simulated magnetization hysteresis loops are shown along with experimental measurements. Numerically obtained magnetic hysteresis loops were used to extract temperature dependent anisotropy constants presented in Fig. 2(b). The simulations were performed within Stoner-Wolfarth model of coherent magnetization rotation for all hard-like hysteresis loops acquired during heating process for both LMOKE geometries. Simulations of hard-axis magnetic hysteresis loops with the characteristic magnetization switching jumps in the vicinity of saturating field were, similarly to our previous report on ferromagnetic Co/Fe(110) bilayers^40^, performed by minimization of the magnetic free enthalpy density as a function of the external magnetic field H:

1 $${\text{G}}\left( {{\text{\varvec{\uptheta}}},{\text{~H}}} \right)={{\text{K}}_1}{\sin ^2}({\text{\varvec{\uptheta}}})+{{\text{K}}_2}{\sin ^4}\left( {\text{\varvec{\uptheta}}} \right) - {\text{H}}{{\text{M}}_{\text{s}}}{\text{cos}}\left( {{\text{\varvec{\upvarphi}}} - {\text{~\varvec{\uptheta}}}} \right),$$

where M_s_ is the saturation magnetization, $\:{\uptheta\:}$ defines the orientation of magnetization with respect to the [001] in-plane direction, ${{\mathbf{K}}_1}$ and ${{\mathbf{K}}_2}$ are the second- and fourth-order effective magnetic anisotropy constants. Angle$~{\mathbf{\varphi }}$ is defined between magnetic field and [001] direction, which means ${\mathbf{\varphi }}$ = 0֯ for H|| [001] and ${\mathbf{\varphi }}$ = 90֯ for H|| [1–10]. It is set to be constant throughout a single numerical loop. We emphasize that the energy minimized during simulation of magnetization reversal process does not include the antiferromagnetic component and corresponding energy term related to FM-AFM interaction. The good agreement between the simulated and experimental magnetic hysteresis loops is a first indication that AFM NiO sublayer does not influence the FM layer magnetic anisotropy significantly. This can also be clearly confirmed by simple visual inspection of magnetic hysteresis loops at 80 K and at 300 K. These loops even at low temperature are almost unaffected by the AFM-FM interaction as they are exchange bias-free and display very small coercive fields for both studied LMOKE geometries. The character of these loops changes between easy- and hard-like which further confirms that observed temperature induced SRT of FM component in NiO/Fe bilayers is solely a consequence of the temperature dependence of MA of Fe.

Results of systematic, temperature dependent LMOKE measurements are summarized in Fig. 2(a) where plots of magnetization in remanent state M_R_ vs. temperature are shown. The solid line in Fig. 2(a) is only a guide for the eye, while each point represents particular LMOKE measurement. For both geometries, M_R_(T) dependencies display some small hysteresis-like windows close to switching temperatures, i.e. for **H** || [001] the value of M_R_(280 K) during cooling procedure is different as compared to M_R_(280 K) during heating of the sample. Similarly, for **H** || [1–10] the values of M_R_(200 K) are different for cooling and heating cycles. However, these features are likely measurement artifacts resulting from minor inconsistencies in temperature readings between cooling and heating measurement series. All hard-like magnetic hysteresis loops corresponding to low level M_R_ ~ 0 were reproduced in simulations, like those already exemplified in Fig. 1. This way, corresponding K_1_ and K_2_ magnetic anisotropy constants used later in our model were determined as a function of temperature, see open symbols in Fig. 2(b). In the temperature hysteresis region of coexistence of easy-like magnetic hysteresis loops makes a determination of anisotropy constants impossible. For this reason, the determined experimental K_1_(T) and K_2_(T) dependencies were approximated by continuous linear functions (solid lines in Fig. 2(b) that provide a description of magnetic anisotropy flow in the whole studied temperature range, also accounting for the range where temperature driven hysteresis occurs in. To investigate the thermal hysteresis we employ experimentally derived, continuous K_1_(T) and K_2_(T) dependencies for numerical calculations of free energy density. The temperature-driven simulation was performed using the following equation:

2 $${\text{E}}\left( {{\text{\varvec{\uptheta}}},{\text{T}}} \right)={{\text{K}}_1}\left( {\text{T}} \right){\sin ^2}({\text{\varvec{\uptheta}}})+{{\text{K}}_2}\left( {\text{T}} \right){\sin ^4}\left( {\text{\varvec{\uptheta}}} \right),$$

which is the (1) equation without Zeeman term and with temperature dependence of K_1_ and K_2_ constants included. Free energy density of the system (2) was then minimized with respect to $\:{\uptheta\:}$ in-plane angle, for each temperature in the 80–350 K range, for both increasing and decreasing temperature branches. Local minimum of energy was found for each temperature and corresponding $\:{\uptheta\:}$ angle defining the magnetization orientation of Fe was determined. It is important to underline that input used for such simulations, namely K_1_(T) and K_2_(T) dependencies, was hysteresis-less as K_1_ and K_2_ constants used in Eq. (2) were determined for heating branch. Nevertheless, the corresponding result of the simulation shown in Fig. 2(c) by solid black line, clearly displays thermal hysteresis of SRT in the temperature range where coexistence of easy-axis LMOKE loops was observed. To explain this, in Fig. 2(e) we present exemplary E($\:{\uptheta\:}$) dependencies at 300 K, 240 K (decreasing T), 170 K and again at 240 K(increasing T). These states are denoted in Fig. 2(c) and (d) by numbers 1, 2, 3 and 4, respectively. One can see that in 1 and 4 states the sample is “before” and “after” temperature induced SRT; at 300 K the minimum of energy is observed only for $\:{\uptheta\:}$ = 90º which means M||Fe[1–10] and for low temperatures the minimum of energy is observed only for $\:{\uptheta\:}$ = 0º which means M||Fe[001]. States 2 and 4 exhibit identical free energy density curves E($\:{\uptheta\:}$) at 240 K. Both local minima are separated by the energy barrier and occupied minimum depends on whether the sample was cooled or heated into that temperature range. That kinetic trapping produces thermal hysteresis of temperature driven SRT.

It should be noted that using only MOKE it is practically impossible to fully reproduce the field-free scenario of temperature SRT, as simulated in Fig. 2(c). For this reason we present the X-ray Magnetic Circular Dichroism (XMCD) and corresponding X-ray Magnetic Linear Dichroism (XMLD) results in Fig. 2(c) and 2(e), respectively. Prior to the measurements, the sample was magnetized in external magnetic field oriented along Fe[1–10] in-plane direction in order to leave it in a single domain state. This ensures non-zero XMCD signal for wave vector of incoming photon beam along easy axis of Fe. Following temperature measurements were performed in remanent state of the sample. Results of normalized XMCD spectra perfectly fit to the simulated solid line in Fig. 2(c) and also correspond very well to bistability borders seen in LMOKE data in Fig. 2(a). Due to exchange coupling between NiO and Fe, the corresponding switching and thermal hysteresis of SRT is also mimicked by corresponding XMLD results shown in Fig. 2(e). As already pointed out, due to small magnetic anisotropy of NiO and well defined strong in-plane anisotropy of Fe, the former one plays rather passive role in the system and its AFM magnetic moments are fully reversible, which means that they are affected by the changes of magnetization orientation in the neighboring Fe. To further confirm this, the example of magnetic field induced switching of both Fe and NiO, represented by element sensitive XMCD and XMLD hysteresis loops, can be found in Supplemental Material (Fig. 4S) respectively. The results shown in Supplementary Material (Fig. 4S) proves that NiO magnetic moments are rotatable and their orientation can be easily switched, either by applying external magnetic field of the order of 100–500 Oe or via the temperature induced SRT in neighboring Fe. Observation of such rotatable AFM spins well agrees with the fact that no exchange bias is observed in NiO(111)/Fe(110). Please note, that field-cooling procedure with starting temperature above NiO Néel temperature does not lead to the onset of exchange bias and also does not freeze the AFM spins of NiO, in contrast with isostructural CoO(111)/Fe(110) system. This means that NiO spins are fully rotatable at low temperature, which can be attributed to much smaller intrinsic magnetic anisotropy of NiO as compared to magnetically hard CoO. However, the AFM-FM coupling is manifested by the small but significant enhancement of coercivity. In Fig. 1a (**H** || [1–10] ) increase of the coercive field is observed with decreasing temperature, from ~ 20 Oe at 300 K to ~ 200 Oe at 80 K, which can be interpreted in terms of rotatable anisotropy^41^ that apparently turns on below Néel temperature of NiO overlayer. Similar effect was observed for easy-axis, square magnetic hysteresis loops, measured on separate sample with Fe thickness far from SRT range (not shown). That temperature induced dependence of coercivity can only be attributed to FM-AFM coupling as for the AFM-free systems the coercive field of Fe(110) films very weakly depends on the temperature. For example the coercive field of 60 Å thick Fe(110) film in W(110)/Fe(110)/W(110) epitaxial stack changes from ~ 30 Oe to ~ 40 Oe as the temperature decreases from 300 K to 10 K^37^. Also, one can note that described increase of coercivity in our NiO/Fe bilayers is not observed for MOKE measurements in **H** || [001] geometry, see Fig. 1b. This further confirms that this effect is not due to simple temperature dependence of Fe coercivity but originates from rotatable anisotropy at the AFM-FM interface.

**Fig. 3:**
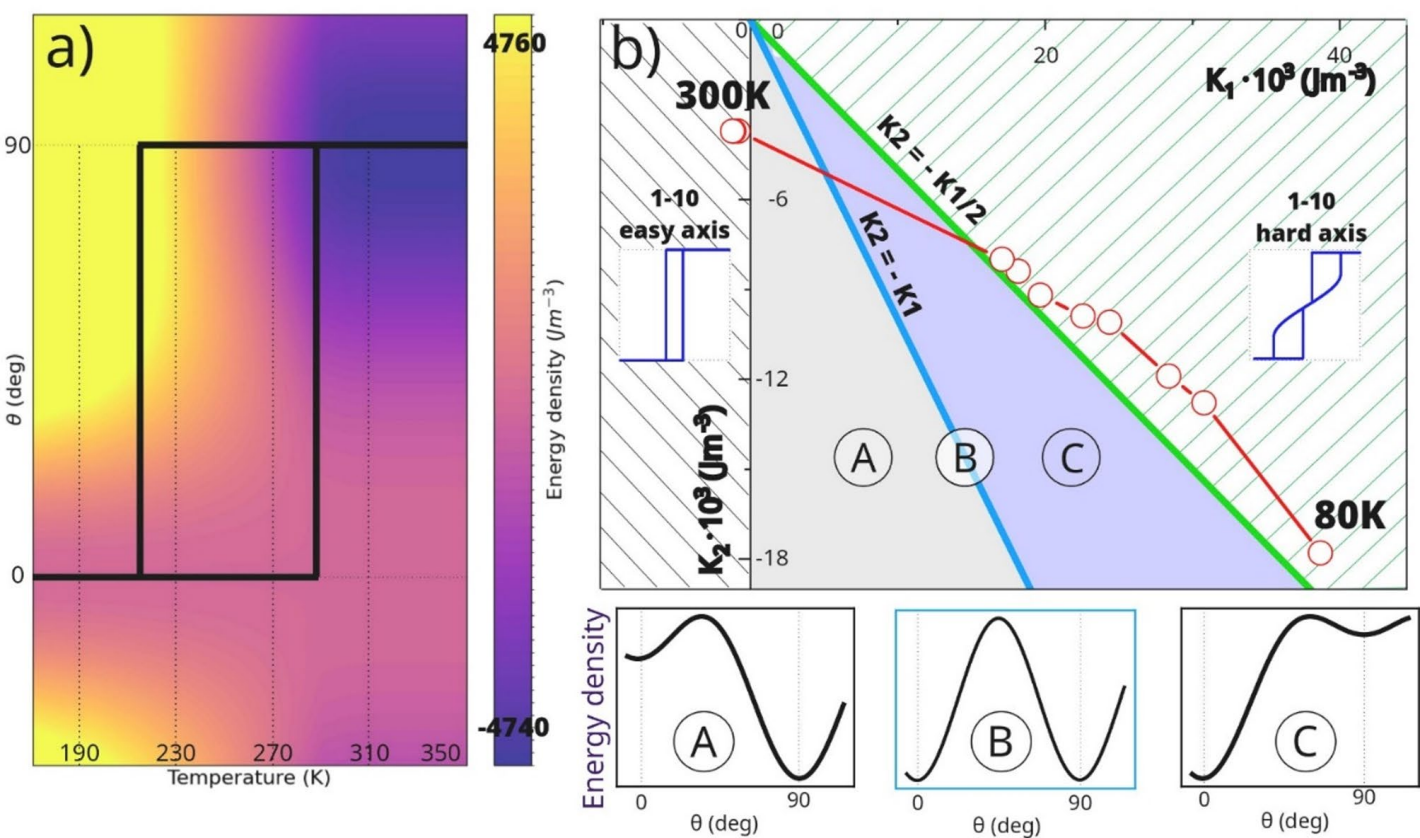
(a) Simulated free energy density spanned across the two-dimensional $\:{\uptheta\:}$-Temperature space. Solid line marks the real magnetic anisotropy flow in NiO/Fe system as determined from MOKE and simulations. (b) Simulated two-dimensional $\:{\text{K}}_{1}\left({\text{K}}_{2}\right)\:$in-plane magnetic anisotropy space for Fe(110) system with NiO/Fe anisotropy flow marked as well. Dashed regions represent bistability phase where hysteresis of SRT can be observed. Insets show local energy vs. $\:{\uptheta\:}$ dependencies and simulated M(H) loops for two characteristic in-plane orientation of external magnetic field H.

The hysteresis can also be noticed in the free energy density landscape on Fig. 3(a). Here, the free energy density at the given temperature reveals an intermediate bistable region where both minima coexist at either 0֯ or 90֯. In Fig. 3(b), the temperature-driven SRT trajectory as determined from data obtained during heating procedure is marked by open symbols on the $\:{\text{K}}_{2}\left({\text{K}}_{1}\right)$ map, showing how the anisotropy constants evolve through the transition. Insets of isothermal E($\:{\uptheta\:}$) dependencies within the bistable region explain why the equilibrium angle $\:{\uptheta\:}$ changes at two distinct critical temperatures despite a single path along which system evolves during cooling and heating cycles.

The observed thermal hysteresis of the spin reorientation transition in NiO(111)/Fe(110) bilayers reflects a delicate balance between magnetic anisotropy contributions in the ferromagnetic layer. Despite the presence of an antiferromagnetic overlayer, the absence of exchange bias and minimal coercivity confirm that the SRT is governed primarily by intrinsic anisotropy changes in Fe. This supports the idea that NiO plays a passive magnetic role, simply following the Fe magnetization without noticeably affecting its energy landscape. The bistable region revealed through experiment and simulation highlight a regime where thermal history dictates the stable magnetization direction. The consistency between simulated and experimental hysteresis loops supports the validity of the modeling and the anisotropy flow approach. As a final remark, a brief comment on the spin reorientation transition, particularly its thermal hysteresis as observed in MOKE experiments, is provided. In MOKE studies, thermal hysteresis can often be overlooked or appear absent due to the influence of the external magnetic field applied during measurements. This field can drive the system into one of the energetically favorable (even metastable) magnetization states, effectively masking the natural coexistence of bistable configurations near the SRT. In contrast, XMCD can probe the remanent magnetic state without external perturbation, allowing for a more direct detection of hysteresis. Because MOKE involves field cycling, it may overwrite the temperature-dependent magnetic history of the sample. As a result SRT may appear as sharp, fully reversible transition without clear hysteresis window, even though bistability exists in the field-free state. This scenario is clearly visible in Figs. 1 and 2(a), where the coexistence of square M(H) loops characteristic of easy-axis behavior is observed near the critical SRT temperatures. If data were acquired for only one LMOKE geometry, one might mistakenly conclude that the SRT is hysteresis-free and extract an incorrect critical temperature.

## Conclusions

In this work, we have demonstrated and systematically analyzed a thermally induced 90º spin reorientation transition in NiO(111)/Fe(110) epitaxial bilayers. The observed spin switching between Fe[1–10] and Fe[001] in-plane directions is shown to exhibit pronounced thermal hysteresis, with clear coexistence of bistable magnetic states in the 210–285 K range. Our results establish that the origin of this hysteresis is rooted solely in the temperature dependence of the magnetic anisotropy (MA) of the Fe layer, with negligible influence from the antiferromagnetic NiO layer due to the absence of exchange bias and the relatively soft magnetic nature of NiO. This is in contrast with our predictions for strongly exchange-biased AFM/Fe(110) bilayers, where depending on magnetic anisotropy of Fe but also on applied cooling procedure a large variety of scenarios is expected (to be published elsewhere). The simplest of the mentioned scenarios is the AFM/Fe with Fe thickness relatively far (but no too far) from critical SRT thickness. Such bilayer, if unbiased, would not reorient its magnetization as the temperature changes. However, exchange bias “frozen” along the hard axis of Fe can potentially rotate Fe spins as EB magnitude increases with decreasing temperature. We expect such effect to be hysteresis-less because it originates mainly from temperature dependence of EB, contrary to what we report in the present article. Here we used temperature-dependent anisotropy constants derived from modeling of MOKE loops and revealed the emergence of hysteresis purely from the interplay of second- and fourth-order anisotropies in Fe. Furthermore, simulations based on these anisotropy constants reproduced the key experimental features, including the thermal hysteresis of magnetization orientation and the bistable magnetic energy profiles. The agreement between MOKE, XMCD, and XMLD data confirms the passive role of NiO in this process and validates the use of NiO/Fe bilayers as model systems for studying pure anisotropy-driven SRT hysteresis in AFM/FM systems.

Our findings not only elucidate the origin of thermal hysteresis in simple ferromagnet/antiferromagnet systems but also provide a framework for engineering thermally tunable magnetic states, potentially relevant for spintronic applications.

## Methods

### Sample preparation

Epitaxial Fe(110) film of 90 Å thickness was grown on an atomically clean W(110) single crystal at room temperature using molecular beam epitaxy (MBE), followed by annealing at 675 K. This produced high-quality Fe film with an atomically smooth (110) surface. Next, a 40-Å NiO adlayer was grown on the Fe(110) surface by reactive deposition of Ni in an O_2_ atmosphere (partial pressure of 1 × 10^–6^ mbar) at room temperature.

### Structural properties

The low-energy electron diffraction (LEED) technique was used to study the surface structure of the Fe(110) and NiO(111)/Fe(110) film. Symmetry of diffraction images observed in LEED confirm the epitaxial (110) oriented Fe films and (111) oriented NiO overlayers. Collected LEED patterns can be found in Fig. 1S of Supplemental Material.

### Magnetic properties

The magnetic properties of the NiO/Fe(110) system were studied *ex situ* using longitudinal magneto-optic Kerr effect (LMOKE) combined with liquid nitrogen cryostat. Temperature dependence of LMOKE magnetic hysteresis loops M(H) was followed for two complementary LMOKE geometries, namely with the external magnetic field H applied either along the Fe[1 − 10] or Fe[001] in-plane direction.

The X-ray Absorption Spectroscopy (XAS) spectra were ex-situ measured at the PIRX end station of National Synchrotron Radiation Centre Solaris in Kraków, in the energy range that covers the L3 and L2 absorption edges of Fe for XMCD studies. Differential spectra for 80 K and 300 K can be found in supplementary material Fig. 2. For XMLD the L2 absorption edge of Ni was followed. In case of AFM NiO, XMLD magnitude is routinely defined by the so called R_L2_ ratio of the two peaks of the L2 absorption edge of Ni in X-ray absorption spectra, please see Fig. 3 in Supplementary Material. We define R_L2_ ratio as the higher-energy peak intensity divided by the intensity of the lower-energy peak. Results of XMLD measurements of the sample in external magnetic field are presented at Fig. 4 of Supplementary Material. XMCD spectra were collected for grazing incidence geometry in which the in-plane sensitivity of XMCD is obtained. Specifically, the wave vector of incoming synchrotron beam was 60º tilted from the sample normal and its projection on NiO(111)/Fe(110) sample plane was along the in-plane Fe[1–10] direction. The direction of the incoming X rays during XMLD measurements was parallel to the sample normal and its linear polarization was parallel to the Fe[1–10] direction (**E** ||[1–10]).

### Simulations

Two kinds of magnetic simulations were performed. The simulated magnetic hysteresis loops were obtained from the minimization of the magnetic free enthalpy density, G(${\mathbf{\theta }},~{\mathbf{H}}$). The simulation of the temperature-driven evolution of the remanent equilibrium state in the NiO(111)/Fe(110) system was based on the local minimization of the free energy density E(${\mathbf{\theta }}$ ,T).

## Electronic supplementary material

Below is the link to the electronic supplementary material.

Supplementary Material 1

## Data Availability

The data that support the findings of this study are available from the corresponding author upon reasonablerequest.
