# Supplementary material for: On the nature of spin reorientation transition thermal hysteresis in NiO(111)/Fe(110) bilayers
Source: Sci Rep. 2025 Jul 1;15:22267. doi: 10.1038/s41598-025-07541-1 (PMC12214746; doi:10.1038/s41598-025-07541-1)
Supplement: Supplementary file 1 — Supplementary Material 1 [file 41598_2025_7541_MOESM1_ESM.pdf]

Supplementary material for:

On the nature of spin reorientation transition thermal hysteresis in NiO(111)/Fe(110) bilayers

E. Świerkosz[1], A. Kwiatkowski[1], M. Szpytma[1,2], W. Janus[3], M. Zając[4], P. Dróżdż[1], E. Oleś[1], A. Kozioł-Rachwał[1], T. Ślęzak[1], M. Ślęzak[1*]

[1] AGH University of Krakow, Kraków, Poland

[2] Elettra - Sincrotrone Trieste S.C.p.A., Basovizza, Trieste, Italy

[3] Institut de Ciència de Materials de Barcelona (ICMAB-CSIC), Campus de la UAB, Bellaterra 08193, Spain

[4] National Synchrotron Radiation Centre SOLARIS, Jagiellonian University, Kraków, Poland

*mislezak@agh.edu.pl

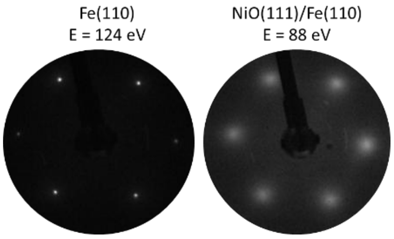

Fig.1S LEED patterns of Fe and NiO in NiO/Fe/W(110) epitaxial system.

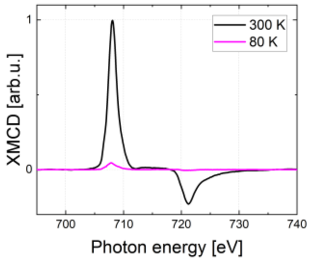

Fig.2S Differential XMCD spectra around Fe L3 and L2 absorption edges at 300 K and at 80 K.

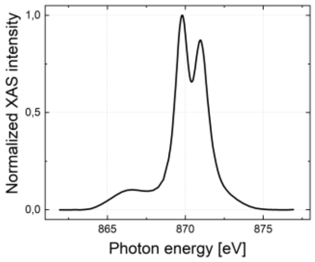

Fig.3S Exemplary XAS spectrum acquired around L2 absorption edge of Ni with two distinct intensity peaks used for R_{L2} ratio definition. R_{L2} ratio is defined as the higher-energy peak intensity divided by the intensity of the lower-energy peak.

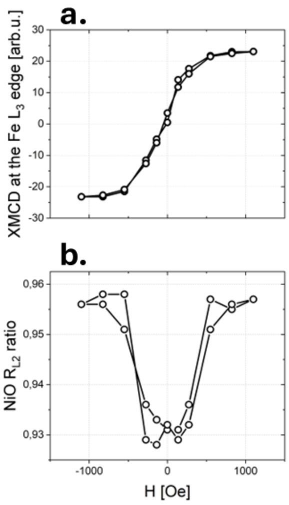

Fig.4S Element-sensitive XMCD (a) and XMLD (b) magnetic hysteresis loop of Fe and NiO, respectively. Characteristic jumps of Fe magnetization during its reversal process are not visible due to small density of acquired experimental points.

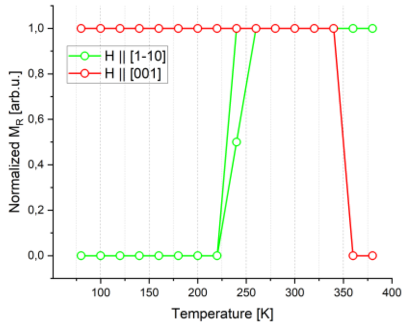

Fig.5S MOKE results for purely ferromagnetic Au(111)/Fe(110)/W(110) SRT system. Normalized magnetization in remanence state M_R as determined from magnetic hysteresis loops, as a function of temperature, for two LMOKE geometries. Thicknesses of Au and Fe sublayers are 20 Å and 80 Å, respectively.
